# Sazgar IoT: A Device-Centric IoT Framework and Approximation Technique for Efficient and Scalable IoT Data Processing

**DOI:** 10.3390/s23115211

**Published:** 2023-05-30

**Authors:** Ali Yavari, Harindu Korala, Dimitrios Georgakopoulos, Jonathan Kua, Hamid Bagha

**Affiliations:** 1 6G Research and Innovation Lab, Swinburne University of Technology, Melbourne, VIC 3122, Australia; 2 School of Science, Computing and Engineering Technologies, Swinburne University of Technology, Melbourne, VIC 3122, Australia; dgeorgakopoulos@swin.edu.au (D.G.); hbagha@swin.edu.au (H.B.); 3 Institute of Railway Technology, Monash University, Melbourne, VIC 3800, Australia; hkorala@swin.edu.au; 4 School of Information Technology, Deakin University, Geelong, VIC 3220, Australia; jonathan.kua@deakin.edu.au

**Keywords:** Internet of Things, approximation, time-sensitive computing, contextualisation, COVID-19, pandemic

## Abstract

The Internet of Things (IoT) plays a fundamental role in monitoring applications; however, existing approaches relying on cloud and edge-based IoT data analysis encounter issues such as network delays and high costs, which can adversely impact time-sensitive applications. To address these challenges, this paper proposes an IoT framework called Sazgar IoT. Unlike existing solutions, Sazgar IoT leverages only IoT devices and IoT data analysis approximation techniques to meet the time-bounds of time-sensitive IoT applications. In this framework, the computing resources onboard the IoT devices are utilised to process the data analysis tasks of each time-sensitive IoT application. This eliminates the network delays associated with transferring large volumes of high-velocity IoT data to cloud or edge computers. To ensure that each task meets its application-specific time-bound and accuracy requirements, we employ approximation techniques for the data analysis tasks of time-sensitive IoT applications. These techniques take into account the available computing resources and optimise the processing accordingly. To evaluate the effectiveness of Sazgar IoT, experimental validation has been conducted. The results demonstrate that the framework successfully meets the time-bound and accuracy requirements of the COVID-19 citizen compliance monitoring application by effectively utilising the available IoT devices. The experimental validation further confirms that Sazgar IoT is an efficient and scalable solution for IoT data processing, addressing existing network delay issues for time-sensitive applications and significantly reducing the cost related to cloud and edge computing devices procurement, deployment, and maintenance.

## 1. Introduction

The Internet of Things (IoT) is the latest Internet evolution that incorporates billions of sensors and other machines (we refer to these as IoT devices or Things) that support the development of IoT-based services and related smart products [1]. Currently, IoT devices (e.g., cameras, smartphones, sensors, smart vehicles, industrial machines, etc.) sense the physical world and send observation data (referred to as IoT data) to IoT-based software services that run in the cloud and edge computers. These computing resources and IoT devices are interconnected via a variety of networks ranging from broadband to low-power IoT networks such as Narrowband IoT (NB-IoT) and LoRa Wide Area Network (LoRaWAN). The unprecedented ability of the IoT ecosystem to observe the physical world and provide valuable information allows IoT-based services to address a magnitude of grand challenges in our society that were unsolvable before due to a lack of critical information from the physical world.

Nevertheless, the full potential of the IoT ecosystem is still far from being fully realised because of the lack of comprehensive solutions that ensure the security and quality of IoT data, improve the reuse of existing IoT devices, provide better support for time-sensitive IoT (TS-IoT) applications, and better support critical industrial applications and industry 4.0 vision.

This paper addresses the challenge of TS-IoT applications (i.e., applications that require their processing to be completed within a specific time-bound, otherwise the produced results will not be useful for the application) [2,3]. TS-IoT applications are relatively common, and they range from preventing traffic accidents and reducing fuel theft in gas stations by preventing vehicles with stolen license plates to refuel to achieving a higher degree of automation control in manufacturing plants, monitoring and managing greenhouse gases, and so forth [4,5,6,7]. In this paper, we focus on a global issue that was critical in mitigating COVID-19 infections: How to use RGB/Infrared/Depth cameras and other sensors deployed in public places to detect citizen COVID-19 lockdown violations and related symptoms and how to do this at a minimal cost?

While it is currently possible to develop an IoT application that utilises IoT devices, such as cameras and sensors to monitor public places, existing IoT approaches for development such as the COVID-19 solution currently follow a ‘device-to-cloud’ architecture that involves IoT devices generating IoT data that are harvested and analysed by cloud-based IoT applications. A later evolution of this architecture includes using ‘nearby’ computers, such as edge computers, located between the IoT devices and the cloud to analyse the IoT device data streams [8,9,10].

Cloud-based data analysis is not ideal for time-sensitive applications because of the significant communication delays involves in sending large and high-velocity data streams for processing in a remote cloud data centre [2]. Edge computing has been introduced to reduce such network delays and related overhead. Unlike traditional cloud-based IoT data analysis, edge computing introduces computers that are located closer to the IoT devices that are used by the IoT applications at hand (the ‘closeness’ is determined by the network delay between the edge and IoT devices). Therefore, the latest trend at this point is focused on edge computing to distribute IoT applications so they can utilise a combination of the available cloud and edge computing resources. Such an approach makes it possible to compute time-sensitive data analysis tasks at the edge, which is ‘closer’ to the IoT devices involved in each data analysis task [8]. However, data processing at the edge is complex, subject to communication delays, and also introduces additional deployment and maintenance costs for edge computing (in addition to the IoT devices and cloud servers). Furthermore, deploying and maintaining (or utilising alternative third-party-provided) edge computers and cloud data centres is expensive, and in some cases, the costs of TS-IoT applications outweigh their benefits.

**Figure 1:**
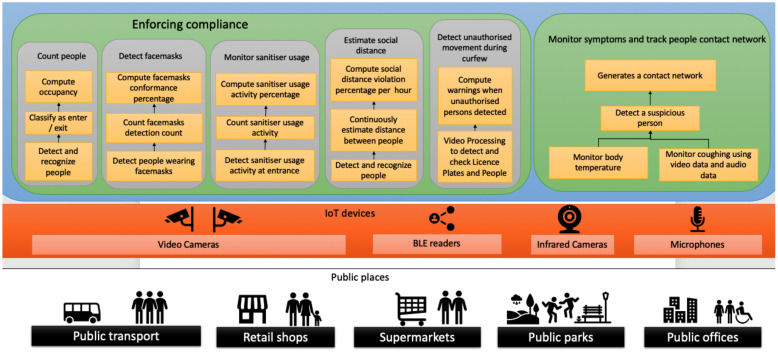
Monitoring government guidance and restriction compliance.

This paper focuses on developing a device-centric IoT framework, referred to as Sazgar IoT (Sazgar is a Persian word meaning adaptable), that can harvest the data and computing power of IoT devices in monitoring citizen compliance with COVID-19 government guidelines and restrictions as shown in Figure 1, e.g., counting people in public transport and other public places such as food stores, detecting and counting the ratio of people that wear face masks, and measuring social distancing. Such a complex application includes many time-sensitive data analysis tasks as compliance metrics must be computed before the situation changes (e.g., people come on board or closer, masks are taken off, etc.). Failure to do so will produce inaccurate information in real-time as public transport occupancy, social distancing, and mask-wearing can change rapidly.

Figure 1 outlines the time-sensitive monitoring of COVID-19 lockdown compliance and symptoms application, including its main tasks and the underlying physical infrastructure and the IoT devices used. The application consists of two main components.

The enforcing compliance component is responsible for monitoring how well COVID-19-related compliance is adhered to by the public during the lockdowns in public areas. To realise this, the application estimates the social distance between people, detects unauthorised movement during curfew, monitors sanitiser usage, detects people who are wearing face masks, and computes occupancy in public places.

The other component of the application is the COVID-19 symptoms monitoring. This component monitors symptoms such as high fever and dry cough of people in public places using various IoT devices and determines potential suspicious COVID-19 patients in real-time. This information is reported to health officials in real time so that the necessary actions can be taken to delay the spread of the virus.

In Section 3, we will further discuss the application.

While video analysis algorithms and related Machine Learning (ML)-based models for identifying objects such as masks, counting people, and measuring their distance are well investigated [11,12,13,14,15], the main challenge in devising this important COVID-19 citizen compliance monitoring application is where and how to compute the related IoT data analysis task in a way that we can meet the application time-bounds so that the compliance measures computed by the COVID-19 citizen compliance monitoring application are accurate.

For example, the COVID-19 citizen compliance monitoring application must compute the number of passengers on a bus or train before the next bus stop, and the number of people in a supermarket by the time any person enters or leaves the supermarket. If this and other time-sensitive applications do not produce information within such application-specific time-bounds, such information is either not useful or problematic, i.e., the COVID-19 citizen compliance monitoring application will conclude that compliance has been achieved while this is not the case, or vice versa.

To address the above challenges, Sazgar IoT considers all related trade-offs, including the resource cost (i.e., purchasing, installing, servicing, and periodically replacing edge computers), the data analysis response time (i.e., the time-sensitive requirements in IoT applications) and the computing resource constraints of IoT devices.

Sazgar IoT meets the time-bounds of time-sensitive IoT applications by (1) analysing all IoT data that are consumed by a time-sensitive IoT application on the IoT devices that generate them; (2) leveraging IoT application-specific time-bounds and descriptions of the computing resources onboard the IoT devices to approximate the IoT data and related analysis task(s) at each device in a way that such approximations of IoT data and/or analysis tasks achieves meeting the time-bounds of each IoT application and effectively manages the utilisation of the onboard computing resources of the devices; and finally, (3) eliminating the costs needed to purchase, deploy, use, and maintain edge computers and cloud data centres.

Existing IoT solutions as shown in Table 1 [8,9,10,16,17,18,19,20,21,22] explore how to best meet the IoT application requirements including Quality of Service (QoS), energy consumption, scalability, latency, security, interoperability, reliability, and cost by utilising the computing resources that are provided by cloud data centres, edge computers and/or IoT devices. However, the problem of meeting IoT application time-bounds by optimising the distribution of TS-IoT applications in such computing resources is NP-hard. In addition, existing research outcomes cannot deal with IoT device and data rate volatility.

This paper explores a device-centric approach that introduces approximation techniques for meeting the time-bounds of TS-IoT applications. Unlike other existing research, this approach does not involve solving NP-hard challenges and can deal with IoT device and data rate volatility.

More specifically, this paper includes the following contributions:

1. An IoT device-centric approach that involves using the computing resources onboard the IoT devices to process the data analysis tasks of each TS-IoT application. This eliminates network delays for transferring large volumes of high-velocity IoT data (e.g., the RGB/Infrared/depth data streams in the COVID-19 citizen compliance monitoring) to the cloud and/or edge computers.
2. Approximation techniques for the data analysis tasks of TS-IoT applications that ensure each task meets its application-specific time-bound and accuracy requirements and at the same time its processing can be completed with the available computing resources onboard the IoT device that harvests the relevant IoT data. IoT task approximation eliminates the complexity of utilising cloud resources and edge computers as well as related cost and time frames for edge computer deployment and cloud resource lease (which are grant challenges in the COVID-19 citizen compliance monitoring).
3. A framework, which we refer to as Sazgar IoT, that combines and implements the above IoT device-centric and approximation approaches. Sazgar IoT can meet the requirements of COVID-19 citizen compliance monitoring (as well as other time-sensitive applications that are outside the scope of this paper).
4. An experimental validation of Sazgar IoT that shows the framework is able to address both time-bounds and accuracy requirements of the COVID-19 citizen compliance monitoring application by utilising only available IoT devices.

The remainder of the paper is organised as follows. Section 2 presents related work. Section 3 explains our COVID-19 citizen compliance application. The device-centric approach in processing TS-IoT applications using approximation is discussed in Section 4. Sazgar IoT framework and the functionality of each engine within the framework are presented in Section 5. Section 6 describes the experimental evaluation for this paper as well as the data analysis and results. Section 7 concludes the paper and outlines the potential future work.

## 2. Related Work

A large amount of work is rapidly building in the area of leveraging technological innovations in digital technologies to combat the COVID-19 pandemic. In this section, we review some of the related work in this area, primarily covering literature and surveys in the areas of using Artificial Intelligence (AI), the Internet of Things (IoT), Unmanned Aerial Vehicles (UAV), Blockchain, and 5G networks in the context of COVID-19. Innovations in these areas have transformed many different applications. The role of these technologies in fighting against COVID-19 is increasingly pronounced in different aspects, ranging from enforcing social distancing and mask compliance to deep analysis of medical imaging.

In a two-part paper, Nguyen et al. [23,24] provided a comprehensive survey of key enabling and emerging technologies for social distancing. The authors covered a wide range of systems and technologies and discussed how existing wireless and networking technologies and AI can enable, encourage, and enforce social distancing practices. In particular, they considered commonly-used communication technologies such as Wi-Fi, Cellular, Bluetooth, Ultra-wideband, ZigBee, and RFID in enforcing social distancing. Their work is followed by a discussion of the many open problems, solutions, and directions in dealing with issues surrounding the enforcement of social distancing, such as early symptom prediction, detection, and monitoring of people in quarantine, tracking people’s movements, density prediction, and contact tracing. More advanced techniques using ML, computer vision, thermal, and ultrasound sensing systems are also discussed. Key issues such as security and privacy while handling personal sensitive data (such as location data) are presented for consideration. The paper also presents possible future responses to a global pandemic using smart infrastructures, smart cities, and intelligent transportation systems enabled by next-generation mobile technologies (5G and beyond).

Hussain et al. [25] surveyed the application of AI and big data in clinical administrations. The authors provided an overview of various techniques driven by AI and big data to be applied to medical information to help medical practitioners and researchers to better understand the virus. Neural systems, classical Support Vector Machine (SVM), and edge significant learning are techniques that are covered in their work. Their work investigated these techniques and demonstrated the effectiveness of using AI and big data to mine the large amount of information available, and also discussed how such techniques can be used in similar medical settings. In a similar vein, Pham et al. [26] also investigated the role of AI and big data in fighting against COVID-19. The authors surveyed a larger body of work, including using deep learning models for early detection and diagnosis; identifying, tracking, and predicting the outbreak; infodemiology and infoveillance; and biomedicine and pharmacotherapy.

In [27], Hossain et al. focused on investigating the use of tactile edge technology and hierarchical edge computing systems enabled by 5G to control and contain infectious diseases. By leveraging the advantages provided by the 5G network, such as its ultra-low latency, high bandwidth, and high reliability, the authors proposed a Beyond 5G (B5G) framework empowered by deep learning (DL) methods for fast processing of chest X-ray images and CT scans, and also enabled a mass surveillance system to monitor social distancing, mask-wearing, and body temperature using smart cameras. The proposed framework has three different in-built DL models, namely ResNet50, Deep tree, and Inception v3. Another aspect of this framework is the use of blockchain to ensure the security of patients’ personal healthcare data. Fog data encryption has also been the focal point of Raghunandan et al. [28] where they used chaotic-map-based encryption in edge computing. The researchers employed several different analyses, such as secret key sensitivity analysis, encryption speed analysis, entropy analysis, and statistical randomness analysis. These analyses were conducted to showcase the security and effectiveness of the algorithm they put forward. The results of the differential attack revealed that the techniques proposed in the algorithm promote diffusion, which signifies resistance against statistical attacks. Such an encryption approach can remarkably address the assurance of data privacy in an IoT environment.

Similarly, Jamshidi et al. [29] also surveyed the applications of DL methods in response to COVID-19, but more from the perspective of diagnosis and treatment. More specifically, the paper covered and illustrated the applicability of DL methods such as Generative Adversarial Networks (GANs), Extreme Learning Machines (ELM), and Long/Short Term Memory (LSTM). The paper summarises some of the medical platforms that use AI and DL methods for deep analysis of medical images and rapid identification of diseases. A particular example is by using a layered approach, starting from the input layer database, selection, image-based techniques, optimisation, and finally, the output that produces the outcome of the final diagnosis. The paper concluded that AI and DL-based platforms can also accelerate the process of diagnosis and treatment of the COVID-19 disease, and discussed the different parameters surrounding DL methods, including properly choosing the inputs and targets of the neural network.

In a separate work, Rahman et al. [30] also investigated a B5G-based tactile edge learning method for handling COVID-19. This framework leveraged edge computing with 5G Radio Access Networks (RAN) by deploying a hierarchical edge computing architecture (ensuring scalability, low latency, and privacy) of data training and analysis. The proposed framework uses a distributed DL approach where each edge node runs its own local DL framework in conjunction with a three-phase reconciliation with the global DL framework in the cloud. Semantics are added to DL models to enable human experts to gain insights and semantic visualisation to facilitate key decision-making activities. The authors implemented a subset of COVID-19 scenarios using the distributed DL framework and demonstrated the effectiveness of the system.

In [31], Nguyen et al. provided a comprehensive review of AI methods in fighting the COVID-19 outbreak and outlined several critical research challenges in furthering AI research to better respond to the COVID-19 pandemic. This paper outlined the key roles, technical advantages, and challenges of AI, ranging from medical image processing, data analytics, text mining and natural language processing and the IoT, to computational biology and medicine. Furthermore, the authors also provided a summary of reliable COVID-19 data sources for research purposes, which can be valuable for the research community. The authors identified future research directions and highlighted specific groups of problems of AI in COVID-19, with the intention of providing the wider community with a comprehensive overview and motivating researchers in utilising AI to combat a global pandemic such as COVID-19.

An important application of IoT in the fight against COVID-19 is the use of drones or Unmanned Aerial Vehicles (UAVs). In [32], Kumar et al. investigated various drone-based systems and architectures, and discussed how these systems can effectively address the different challenging scenarios brought about by the COVID-19 pandemic. The proposed architecture involves the use of wearables in Body Area Networks (BANs) and implemented drone-based systems to collect a large amount of data across a large area. These data collection and analysis can be applied to use cases such as large-area sanitisation, thermal image collection, and patient identification within a short period of time. The work also outlined open questions and research challenges for drone-based technologies.

Chamola et al. [33] also provided a comprehensive survey on the use of UAVs, IoT, blockchain, AI, and 5G to mitigate the impact the COVID-19. The authors discussed in detail the Internet of Medical Things (IoMT), which is the collection of medical devices and software applications that are connected to the larger healthcare systems. The IoMT is increasingly prevalent with an increasing number of mobile devices being equipped with Near Field Communication (NFC) and Bluetooth capabilities. IoMT can enable the monitoring of patients from a remote location, the tracking of medical orders, and the use of wearables to transmit healthcare data. Similar to IoT, IoMT enables the effective collection, analysis, and transmission of large amounts of healthcare data. The authors also discussed the roles of drones, robots, autonomous vehicles, wearables, telemedicine, mobile apps, blockchain for distributed databases, and 5G for thermal imaging. The different types of drones, such as multi-purpose drones, thermal imaging drones, announcement drones, disinfectant drones, surveillance drones, and medical drones are also discussed.

Gupta et al. [34] discussed in detail how blockchain can be used to facilitate UAVs in response to the COVID-19 pandemic. The authors proposed multi-swarm UAVs with 5G systems that aim to reduce human intervention (and reduce the risks of infections) while performing various critical operations. The UAV system utilised the advantages of 5G to send a large amount of data to ground stations in real-time, which enabled 5G’s high bandwidth, high availability, and low latency properties. The blockchain component of the framework addresses the security and privacy issues when sending sensitive data across the network, which can be coupled with the upcoming innovations in 6G for using Terahertz (THz) frequency bands, the virtualisation of link and physical-layer protocols, and the softwarisation of communication infrastructure. Their studies showed that the proposed system achieved improved performance in terms of processing delay, packet losses, and throughput when compared with existing 4G/5G systems.

In [35], Siriwardhana et al. provide an overview of how 5G and IoT can be fully utilised in the battle against the COVID-19 pandemic. More specifically, the authors identified seven use cases, along with their technical requirements, that can benefit from the latest advances in 5G and IoT, namely telehealth, contact tracing and self-isolation, online education, retail and supply chain, smart manufacturing and factory automation, e-government and media, e-tourism, and entertainment. The paper also discussed how the advancements and developments in these areas can benefit society in a post-pandemic world.

The state-of-the-art IoT solutions discussed in the related work and presented in Table 1 primarily concentrate on implementing device-to-cloud IoT architecture. This preference stems from the advantages offered by cloud-based IoT architecture, such as scalability, advanced analytics, data storage and management capabilities, integration with external services, and remote monitoring and management features. However, it is important to acknowledge that relying solely on a cloud platform can have a detrimental effect on meeting the time constraints of time-sensitive IoT (TS-IoT) applications.

The process of transferring data from the IoT device layer to the cloud inherently introduces latency, which can vary depending on the type of data and the capacity of the communication network. This latency, as well as the possibility of data loss during data transfer from IoT devices to the cloud, can significantly impact IoT applications where meeting time constraints is of utmost importance. In such scenarios, a device-centric architecture such as the Sazgar framework can address these issues.

The Sazgar framework utilises approximation techniques to perform data processing at the first layer of the IoT architecture, which is the IoT device layer. By adopting this approach, the negative effects of latency and data loss on TS-IoT applications can be mitigated or eliminated.

## 3. Scope and Requirements of COVID-19 Symptoms and Compliance Monitoring Applications

The effective monitoring of citizens’ compliance with COVID-19 lockdown restrictions is important for governments and municipalities across the world as this can provide important information that they need to ease or tighten lockdown restrictions, as well as formulate related policies and action plans for mitigating COVID-19 transmission. However, to be effective, COVID-19 citizen compliance monitoring must be both accurate and timely. More specifically, IoT applications for monitoring citizen compliance to COVID-19 restrictions must accurately and in a timely manner sense and compute the following high-value information for citizen activities in public places, such as buses, train carriages, bus stops, playgrounds, and food stores: (1) the real-time occupancy of public places; (2) social distancing in public places; (3) wearing masks; (4) COVID-19 symptoms, i.e., high body temperature and coughing; and (5) related citizen contact networks (e.g., the network of all passengers who happen to ride the same bus or the citizens that shopped in the same supermarket) without violating citizen privacy. In this paper, we consider an IoT application for monitoring COVID-19 citizen compliance. More specifically, we consider how to accurately and in a timely manner sense and compute the following:

- Social distancing: The World Health Organisation (WHO) and governments around the world recommend that during the pandemic citizens should maintain at least 1.5 m social distancing in public places [36]. Physical greetings such as handshaking, hugs, and kisses should be avoided. The COVID-19 application can use image processing and the relevant classifier on RGB camera data to estimate the distance of people in a public place.
- Wearing mask: The WHO, and some governments around the world, require health workers and citizens to wear a mask in the workplace or in public places [37,38].
- Curfew: Some governments around the world have imposed city-wide curfews to control the pandemic. For example, Melbourne in Australia instituted a 9 p.m. to 5 a.m. curfew for several weeks.
- Visiting hotspots: Some governments have declared hotspots in areas with a disproportionately greater than usual number of COVID-19 infections. This requires tracing citizens entering and leaving hotspots.
- People counting: Government restrictions often include limits on the number of people in specific public spaces, e.g., supermarkets.
- Symptoms: Monitoring COVID-19 symptoms (e.g., fever and coughing) is common in public places such as stations, schools, and workplaces.
- Contact networks: Many governments recommend that citizens use contact tracing applications on their mobile phones to assist in COVID-19 mitigation.

To provide COVID-19 compliance monitoring, the above citizen activities, symptoms, and contact networks must be detected within specific time-bounds and with specific accuracy to ensure the validity of such information.

Table 2, provides sample detection time-bound and corresponding accuracy required for detecting each of the above citizen activities and symptoms [37,39,40,41,42,43,44,45]. Although the detection accuracy for some of the activities such as people counting is relatively low, this data is only used as a basis of framework presentation. If detection of any activity or symptom occurs after its time-bound or with less accuracy than specified in Table 2, the COVID-19 compliance monitoring is ineffective.

As described in Table 2, the main challenge in this IoT application is to achieve the appropriate balance between timelines and accuracy, in sensing and computing the above citizen activities and symptoms.

## 4. Device-Centric Approach in Processing TS-IoT Applications Using Approximation

Sazgar IoT proposed in this paper can meet the requirements of the COVID-19 compliance and symptoms monitoring application as well as other similar TS-IoT applications. To achieve this, Sazgar IoT incorporates a combination of an IoT-device-centric architecture with approximation-based IoT data analysis techniques. Data approximation is defined in Definition 2.

**Definition** **1.** *Contextualisation is defined as “the process of filtering, aggregating, and inferring IoT data by using relevant information to the applications using the data” [1].*

**Definition** **2.** *Data Approximation is using contextualisation to reduce data in terms of volume, variety, and velocity to deal with computational resource constraints while satisfying the application requirements (e.g., time-bound, accuracy, etc.).*

To further explain these contributions, we first describe how Sazgar IoT processes TS-IoT applications in Section 4.1. Section 4.2 presents the device-centric architecture of Sazgar IoT, provides an example of a TS-IoT application execution in an IoT device, and discusses its benefits and limitations. Finally, in Section 4.3, we present the principles of approximation-based data analysis and discuss how such techniques address the limitations of device-centric architecture we introduced in Section 4.1.

### 4.1. TS-IoT Application Processing in the Device-Centric Framework

**Figure 2:**
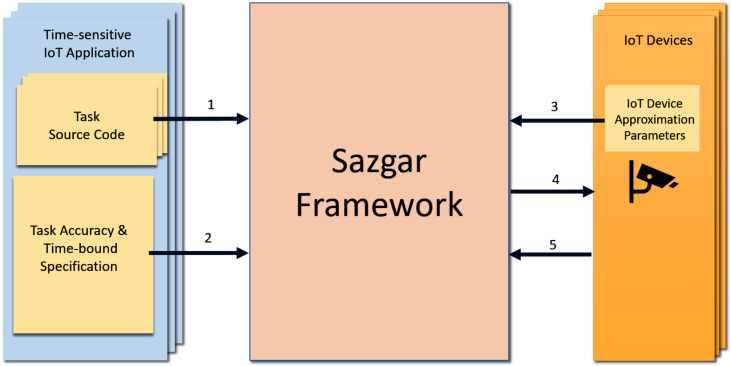
Device-centric approach in processing TS-IoT applications in Sazgar IoT.

Figure 2 shows how Sazgar IoT processes TS-IoT applications. More specifically, as we discussed in Section 3, TS-IoT applications are submitted to Sazgar IoT as a set of (possibly interdependent) tasks, which include the task source code that was provided by the TS-IoT application developers, and a TS-IoT application accuracy and time-bound specification file that defines the application’s accuracy and time-bound requirements for each of the tasks that comprise the application. Figure 2 shows the submission steps of the accuracy and time-bound tasks’ specification files of the TS-IoT application by arrows labelled with ‘1’ and ‘2’, respectively. Table 2 in Section 3 provides an example of the accuracy and time-bound requirements of the COVID-19 symptoms and compliance application. The accuracy and time-bound specification format for the TS-IoT application are discussed further in Section 5.1.

IoT device providers that make IoT devices available to Sazgar IoT independently submit their approximation parameters to the framework. Figure 2 depicts the submission of the approximation parameters by IoT device providers with the arrows labelled ‘3’. This can take place at any time. However, the Sazgar IoT framework considers only the IoT devices whose approximation parameters have been submitted by the point in time steps ‘1’ and ‘2’ as completed. The details of the IoT device approximation parameter format are presented in Section 5.2.

When a TS-IoT application task cannot meet its TS-IoT application requirements, the Sazgar IoT framework uses the approximation techniques we discuss in Section 4.3 to tailor the IoT device parameters and/or the task’s sources code in a way that the task can meet its time-bound and accuracy requirements when it is executed in its target IoT device. The submission of the tailored approximation parameters/task code is depicted in Figure 2 by the arrow labelled ‘4’. Approximation techniques are explained later in this section. The IoT data that results from the task execution in the IoT devices (which we refer to them as task reports) to the Sazgar IoT framework is shown with the label ‘5’ in Figure 2.

### 4.2. Device-Centric Architecture

As illustrated in Figure 2, Sazgar IoT uses the available IoT devices that generate the IoT data required by each TS-IoT application to process its tasks.

This device-centric architecture eliminates all IoT data harvesting-related communication delays, and it may only require concise task reports to be sent to a designated IoT device or the cloud. To explain this further, consider the ‘People counting’, ‘Wearing masks’, and ‘Social distancing’ tasks of the COVID-19 symptoms and compliance appliance application in Table 2. Sazgar IoT meets the time-bound and detection accuracy requirements of these tasks by harvesting and analysing IoT data directly from the IoT sensors connected to the IoT device. This data is utilised to compute the results for each task.

The device-centric IoT architecture is cheaper and faster to implement as it does not require edge computers to harvest IoT data. More importantly, it is not subject to related network delays from remote harvesting of IoT data. Therefore, the device-centric architecture of Sazgar IoT allows meeting the time-bound requirements of the COVID-19 compliance application in Table 2 by:

- avoiding the transmission of large, high-velocity IoT data streams to the cloud and/or edge computers that are required by existing device-to-cloud and device-to-edge-to-cloud approaches;
- eliminating related network delays (e.g., some cloud-based IoT platforms suffer from 300+ ms network delays that make meeting the requirements in Table 2 impossible);
- not requiring a large-scale (e.g., city or country-wide) deployment of edge computers that is very costly and will take significant time to implement.

### 4.3. Principles of Approximation-Based Data Analysis

The main benefit of the device-centric architecture employed by Sazgar IoT is that it eliminates all communication delays in the IoT data harvesting performed by TS-IoT applications. However, IoT devices typically have limited onboard computing resources (i.e., microprocessors with relatively small memory and storage) that may not be enough to compute their stateful or even stateless data analysis in such a way that TS-IoT tasks meet their time-bounds and accuracy requirements. To address this challenge, Sazgar IoT incorporates innovative approximation techniques for TS-IoT tasks.

The approximation techniques proposed in this paper take as input: (1) the task source code and time-bound specification files of each TS-IoT application, and (2) the descriptions of available computing resources in the IoT device that is targeted for processing each task. The approximation techniques then use this information to tailor the IoT data harvesting rate or/and accuracy so that the TS-IoT application tasks can be computed within its required time-bound and accuracy. More specifically, when a TS-IoT task *t* needs to be processed at the target IoT device *d*, Sazgar IoT uses either or both of the following approximation techniques:

- IoT device reconfiguration: This technique reconfigures the IoT device *d* so that the computing resources needed to harvest the IoT data harvested by *t* are less than the resources that are currently available in *d*;
- IoT data harvesting method substitution: This technique generates an IoT data harvesting method for *t* and it substitutes the application-provided IoT data harvesting method in *t* so *t* uses less computing resources than those available in *d*.

To provide examples of both these approximations techniques, please consider the task that detects social distancing in Table 2. The IoT device reconfiguration technique adds a configuration method to this task that reduces the frame rate and/or resolution of the IoT data from the onboard camera(s) so that the IoT data harvested for the social distancing task can be processed with the available computing resources there and at the same time distancing violations involving citizens in the camera view is detected with 65% accuracy. The IoT data harvesting method substitution technique achieves a balance between the computing resource requirements of the social distancing in Table 2 and the available computing resources in the target IoT device by substituting the IoT data harvesting method in the social distancing task with a method that only measures the distance between a specific number of samples of citizens per minute (e.g., as they are entering and leaving the bus).

To automatically determine the degree of approximation required for each specific task and IoT device pair, we use contextual information (e.g., the available computing resources in each IoT device and TS-IoT application requirements, such as those in Table 2. Specific parameters used to perform the approximation include:

- IoT device computing resource parameters, including the IoT device’s CPU and RAM usage;
- TS-IoT task IoT data harvesting parameters, including resolution of the video, colour profile (e.g., RGB, grey-scale, black-and-white), frame rate, and classification algorithm (e.g., depth or RGB).

Sazgar IoT uses these parameters to estimate (1) the accuracy of the task’s results (i.e., the passenger counting accuracy in the bus), and (2) the CPU and RAM usage in the target IoT device.

**Figure 3:**
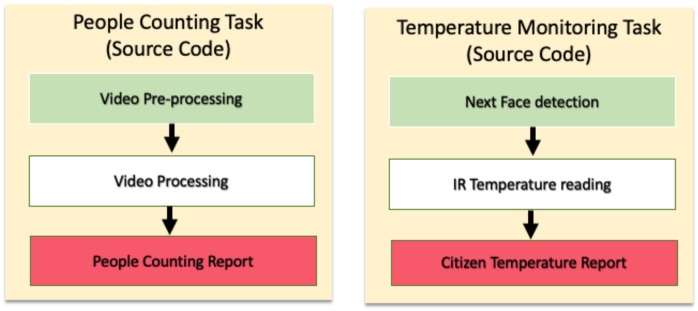
Example of tasks and sub-tasks in TS-IoT applications.

Each TS-IoT application (e.g., IoT COVID-19 compliance) comprises several tasks (e.g., people counting, social distancing, etc.). Each of these tasks has a corresponding source code that is an executable description of the tasks and is provided by the TS-IoT application. These tasks can be divided into sub-tasks (e.g., video pre-processing) as illustrated in Figure 3.

Each of these sub-tasks may need to perform one of the following: consume IoT data from heterogeneous IoT devices, perform data processing ranging from basic stream processing to resource-intensive ML and statistics, manage the data queues required for stateful data analysis, and produce information that is used by other sub-tasks or reported to the users.

**Figure 4:**
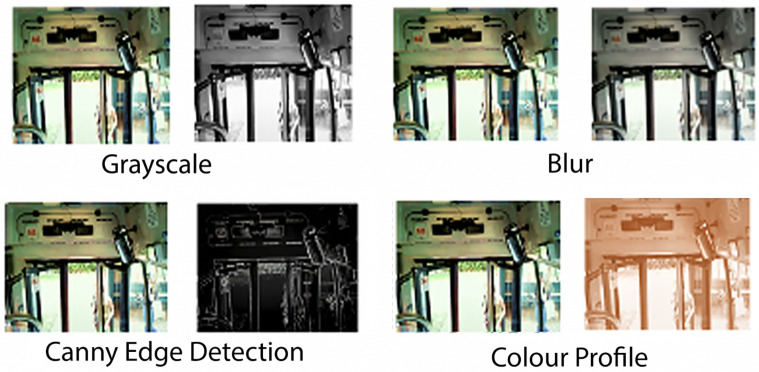
Example of pre-processing algorithms investigated in COVID-19 application.

For example, in the people counting task we have video pre-processing, video processing, and people counting reporting tasks. Video processing involves running the Haar Cascade classifier [46,47] on the video to detect human heads that need to be counted. Video pre-processing is for preparing the data for the classifier. A few examples of pre-processing sub-tasks are illustrated in Figure 4. This video pre-processing is required for the classifier to reduce the complexity of the processing of the video. Reporting involves the mathematical summation of the heads and reporting the final result (i.e., the number of people).

The sub-tasks are not necessarily the same for all the tasks. For example, the temperature monitoring task will involve next face detection, infrared temperature processing, and providing citizen temperature reports in contrast with video pre-possessing, video classifier, and people counting report in people counting task as shown in Figure 3. All of these sub-tasks rely only on IoT devices to complete all required IoT data analysis tasks. This IoT-device-based analysis eliminates network delays from transferring high volume and velocity IoT data streams.

The TS-IoT application should provide the source code to Sazgar IoT to enable capturing accuracy, resource consumption, and time required to perform each of these sub-tasks. Sazgar IoT is responsible for performing the approximation and contextualisation to make sure the IoT application will meet the time-sensitivity and accuracy requirements.

The Sazgar IoT framework is described in detail in Section 5.

## 5. Sazgar IoT Framework

**Figure 5:**
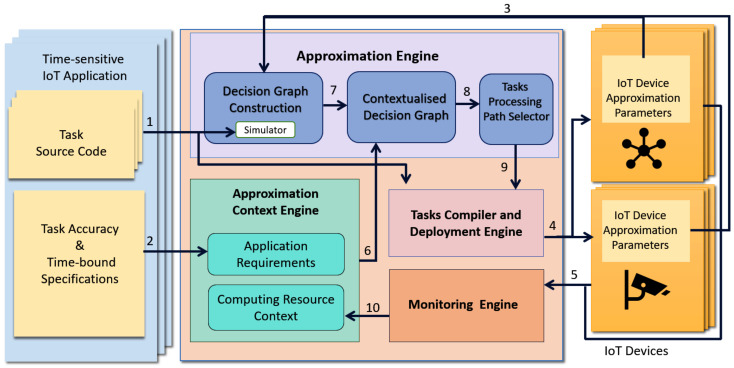
Sazgar IoT framework.

In this section, we will discuss the Sazgar IoT framework. As illustrated in Figure 5, the Sazgar IoT framework consists of four main engines as follows:

- Approximation Context;
- Approximation;
- Task compiler and Deployment;
- Monitoring.

In the following section, we discuss the purpose and functionality of each of these engines. More specifically, the Approximation Context engine is presented in Section 5.1. The Approximation and Task Compiler and Deployment engine are discussed in Section 5.2 and Section 5.3, respectively, while the Monitoring engine is introduced in Section 5.4.

The IoT application tasks can have input from one or more IoT devices. In this paper, we assume the data is coming from one IoT device. However, it is possible to decompose the tasks into sub-tasks and distribute them among different IoT devices (e.g., the IoT devices that are contributing the majority of the data for the task), which is outside of the scope of this paper.

### 5.1. Approximation Context Engine

The Approximation Context engine is responsible for collecting the application-specific requirements of the TS-IoT application (we refer to this as application context) in the form of time-bound and accuracy pairs for each of the application’s data analysis tasks and sending these specifications to the Approximation engine (discussed in Section 5.2) that (if needed) uses the specified time-bound and accuracy of each task to approximate it.

A TS-IoT application consists of several data analysis tasks. For example, the COVID-19 IoT application may consist of data analysis tasks such as social distance monitoring tasks, face masks wearing monitoring tasks, passenger counting tasks, etc. Such data analysis tasks have an accuracy and a time-bound requirement that need to be met during the execution.

**Figure 6:**
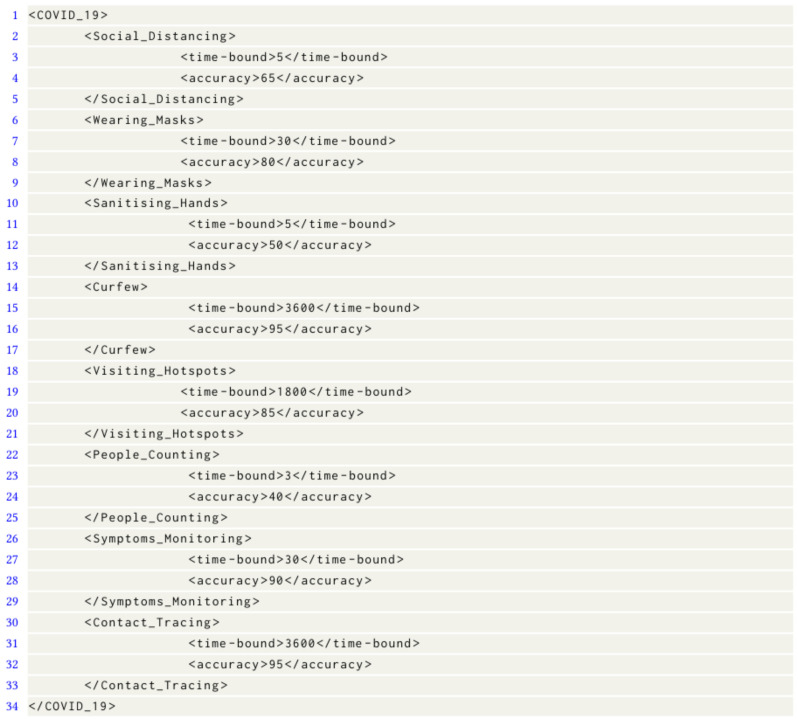
Accuracy and time-bound specifications for COVID-19 citizen compliance monitoring application.

For example, the requirement of the COVID-19 citizen compliance monitoring application in Table 2 is captured in an application specification file that is illustrated in Figure 6. It includes a time-bound and accuracy pair for each of the tasks in this application. The Approximation Context engine expects application specification files to be in XML format as illustrated in Figure 6 for the COVID-19 citizen compliance monitoring application tasks. Please note that the specified accuracy in Figure 6 is in percentage and the time-bounds are in seconds. Furthermore, each data analysis task comprises a set of actors that work together to complete the data analysis. For example, a passenger counting data analysis task may involve actors such as video pre-processing, video processing, and people count reporting.

In addition to the above specifications of TS-IoT applications, the Approximation Context engine periodically collects information about the available computing resources (i.e., the CPU usage, memory usage and execution times, etc.) that is provided by the Monitoring engine (discussed in Section 5.4) and forwards this information (we refer to this as computing resource context) to the Approximation engine, which uses this information to keep task approximations computable within the available computing resources at each relevant IoT device. More specifically, the computing resource context includes: (1) the available computing resources at the relevant IoT device (e.g., available processing, memory, and storage), and (2) the resources consumed by each running data analysis task (e.g., CPU usage, memory usage and processing time) for the same or other TS-IoT applications.

This solution allows the Approximation Context engine to deal with IoT data rate and device volatility. In particular, in the presence of more volatility, the engine increases the rate of collecting computing resource context and forwards it to the Approximation engine. In the rest of this paper, we refer to both application context and computing resource context as contextual information [1].

### 5.2. Approximation Engine

As its name implies, the Approximation engine uses the contextual information that is provided by the Approximation Context engine to tailor tasks of submitted TS-IoT applications in a way that each data analysis task can be fully executed with the computing resources available in a target IoT device while meeting its specified time-bounds and accuracy.

**Figure 7:**
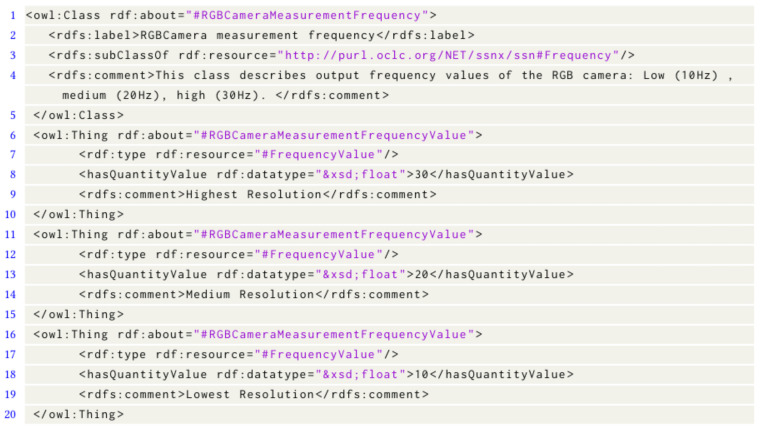
IoT device approximation parameters.

The Approximation engine receives two inputs including (1) Contextual information provided by the Approximation Context engine and (2) IoT device approximation parameters provided by the IoT device. IoT device approximation parameters are the parameters that can be modified to perform approximation. For example, Figure 7 shows that the IoT device has the capability to support three frame rates per second. IoT device approximation parameters are described with Semantic Sensor Network (SSN) ontology [48] and are described in more detail in Section 5.2.1. The Approximation engine accomplishes this by performing the following:

1. Constructs the decision graph that will be described in Section 5.2.1. The decision graph construction aim is to capture IoT device approximation parameters provided by IoT devices and will use those parameters to provide resource consumption for all the possible combinations of IoT device approximation parameters as well as expected accuracy and time-bounds for computing the tasks with those corresponding parameters.
2. Contextualise the decision graph that will be described in Section 5.2.2. It uses the constructed decision graph as an input to construct the contextualised decision graph. This contextualised decision graph is a contextual filter of the decision graph [49]. The output of this technique is a subset of the constructed decision graph where it only includes paths that can be computed with the available computing resources (provided by Computing Resource Context) and also satisfy the application specifications in terms of accuracy and time-bounds. For example, for the COVID-19 citizen compliance monitoring application we will have only paths that can satisfy the accuracy and time-bounds mentioned in Table 2 while they can be computed with the currently available computing resources.
3. Determining the most suitable Task Processing Path that will be described in Section 5.2.3. This technique will use a contextualised decision graph as an input and will select the paths that can achieve the highest accuracy to be provided to Task Compiler and Deployment engine.
4. Uses computing resources context updates from the approximation context engine to dynamically contextualise the decision graph during the run-time.

In the following sections, we present the techniques we developed for constructing contextualised decision graphs and determining the most suitable Task Processing Paths.

#### 5.2.1. Decision Graph Construction Technique

**Figure 8:**
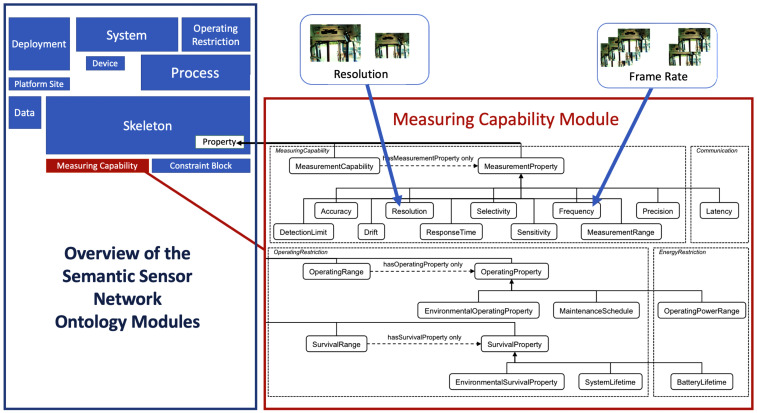
IoT device approximation parameters conceptual description utilising the Semantic Sensor Network Ontology.

This technique constructs a directed acyclic graph, which we refer to as a decision graph, before the start of the application execution. To facilitate this, each available IoT device provides IoT device Approximation Parameters to the Approximation engine. These specifications describe the various approximation parameters such as IoT data type (e.g., RGB, Depth), number of frames (e.g., 10 frames), and so forth involved in the TS-IoT application. The “IoT device approximation parameter” follows SSN ontology concepts [48]. IoT device approximation parameters in this paper are described in the ‘MeasuringCapability’ module of the SSN as illustrated in Figure 8.

Figure 7 represents an example of IoT device approximation parameters describing that the device has an RGB camera that supports three different frame rates including Low (10 fps), Medium (20 fps), and High (30 fps).

The decision graph construction technique includes a cloud-based simulator that can be configured according to the system configurations of the underlying IoT device by the users of Sazgar IoT. As noted before, each data analysis task of the TS-IoT application comprises a set of actors. Each actor of the data analysis task has a corresponding sample actor (i.e., an actor that can be simulated in the simulator) and a source code actor (i.e., an executable unit of the framework that is a compiled source code). By using this simulator and IoT device approximation parameters, before the actual deployment and execution of the IoT application, first, we simulate the sample actors of the data analysis tasks under each of the IoT device approximation parameters specified in the file and observed. Each of these simulations under different IoT device approximation parameters will lead to an observation of accuracy, resource consumption (observed from the simulator), and total application execution time. We refer to these observations as Task Processing Paths. After the simulations, by using these Task Processing Paths, the technique constructs a contextualised decision graph.

#### 5.2.2. Contextualised Decision Graph Technique

The contextualised decision graph would define the approximation of the IoT processing for each application data analysis task (i.e., video frame rates, video resolution, etc.) which leads to different resource usages, accuracy levels, and total application execution times. As we discussed earlier, each data analysis task comprises a set of actors and each actor has its own IoT device approximation parameters (e.g., sample actor variation for frame size IoT device approximation parameter may involve 10 frames actor, 20 frames actor, and 30 frames actor). This is only performed at the beginning of the actual application deployment. The constructed decision graph will then be used to identify the most suitable Task Processing Path that meets the time-bound requirements of the application based on the contextual information.

The decision tree construction technique has the capability to update the Task Processing Paths based on the resource consumption information of the executed actors in cases where the observations (i.e., resource consumption, total application execution time) are not matching the simulation output. To realise this, the decision tree construction technique utilises the computing resource context information (mainly the resource consumption information of the actors), which is periodically sent by the approximation context engine. In addition, this technique can add to the Task Processing Paths by executing sample actor variation in IoT device, edge, or cloud to estimate accuracy, computing resource context, and time-bound for the actors not provided by the simulator.

#### 5.2.3. Task Processing Path Selector Technique

This technique is responsible for selecting the most suitable actor processing for each of the data analysis tasks that can meet the time-bound requirements of the TS-IoT application. A Task Processing Path is a single end-to-end path of a data analysis task in the contextualised decision graph. For example, in our use case, the video pre-processing actor (e.g., grey-scale) → video processing actor → people count report actor is a sample Task Processing Path of the passenger counting data analysis task. Before the actual deployment of the TS-IoT application, the Task Processing Path selector receives the contextualised decision graph that was constructed with the help of the simulator. Then, the Task Processing Path selector technique uses the application requirements context received from the approximation context engine to select the most suitable Task Processing Path that leads to an observation of the highest accuracy within the time-bound requirements specified in the application requirements context for each individual task. The selected Task Processing Paths will be forwarded to the Task Compiler and Deployment engine which will then manage the deployment of the IoT application into the IoT device. This will be discussed in Section 5.3.

During the run-time of the TS-IoT application, the approximation context engine periodically collects computing resource context from the monitoring engine, and this information is sent to the Approximation engine. Whenever the observed total application execution time is beyond the time-bound requirements of the TS-IoT application, the Task Processing Path selector disables the existing Task Processing Path and dynamically finds a suitable Task Processing Path that has the next highest accuracy. In order to achieve this, the engine uses a “Dynamic decision graph-based algorithm”, which is discussed in Section 5.2.4. Then, this Task Processing Path is forwarded to the Task Compiler and Deployment engine for a redeployment.

#### 5.2.4. Dynamic Decision Graph-Based Algorithm

**Figure 9:**
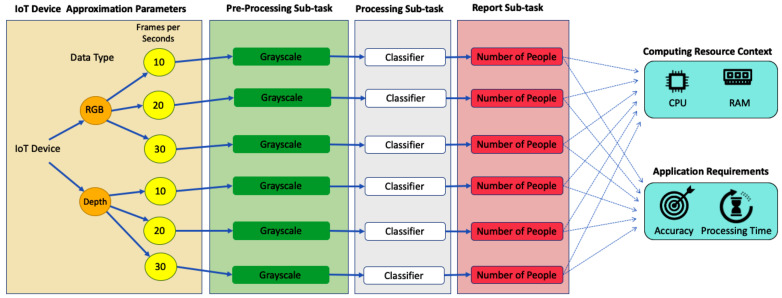
Approximation Decision Tree Graph.

In our proposed solution, we observe the resource consumption and execution time of the actors under different IoT device approximation parameters. Each execution of an atomic process will lead to an observation of execution time and consumed resources. We then use these observations to update the constructed decision graph, which defines the approximation of the IoT processing for each application task (i.e., video frame rates, video resolution, type of classification algorithm used, etc.), which leads to different resource usages, accuracy levels, and execution times [Figure 9].

The constructed decision graph can be used to identify the most suitable Task Processing Path (i.e., suitable frame rate, video resolution) that would help meet the application’s time-sensitive requirements while (at least) achieving an accuracy level defined by the application.

In our proposed Task Processing Path selection algorithm, we dynamically identify the most suitable Task Processing Path, whenever the contextual information changes. The proposed algorithm continuously checks whether the contextual information is updated or not. Whenever the contextual information is updated, it collects that contextual information and passes it to the “Approximate-IoT-Data” function. The approximate-IoT-Data function is executed in the Approximate engine. The function first takes the approximation decision graph, which was constructed initially with the simulation. Subsequently, the algorithm contextualise the decision graph using the computing resource context. Here, the Task Processing Paths in the decision graph are updated based on the resource consumption information of the source code actors in cases where the observations (i.e., resource consumption, total application execution time) do not match the simulation output. After that, the algorithm finds the most suitable Task Processing Path that yields the highest accuracy within the time-bound requirements of the application using the application requirements context. This “actor-process-path” is then forwarded to the Actor construction engine via the Create-Actors function. The Create-Actors function constructs the source code actors and deploys them into the IoT devices. Our dynamic Task Processing Path selection algorithm is shown in Algorithm 1.
**Algorithm 1:** Pseudo-code for dynamically deciding the Task Processing Path, creating corresponding actors, and deploying them in IoT devices
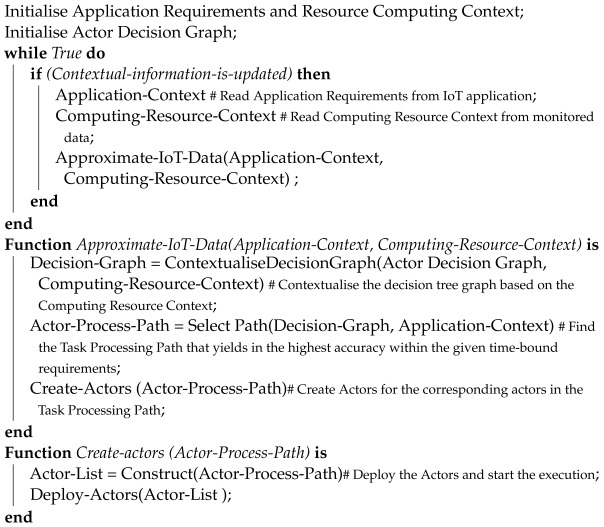


### 5.3. Task Compiler and Deployment Engine

This engine is mainly responsible for constructing the source code actors(i.e., executable units of the framework that are compiled source codes) that can run in the IoT devices and deploying them into the IoT device. To achieve this, the engine accepts the Task Processing Path selected by the approximation engine and constructs the corresponding source code actors for each actor in the Task Processing Path. Finally, the engine deploys the corresponding source code actor to the IoT device and starts its execution. Whenever the engine receives an updated Task Processing Path, it stops the execution of the running source code actor in the IoT devices and deletes them. After that, it constructs new source code actors according to the updated Task Processing Path and deploys them into the IoT device. The IoT device is responsible for executing each source code actor and collecting the data. In the scope of this paper, there is no distribution of the tasks between the IoT devices.

### 5.4. Monitoring Engine

This engine is responsible for continuously monitoring the performance metrics of the underlying IoT device and source code actors. The monitoring engine periodically collects performance metrics such as available CPU, memory, and storage from the underlying IoT device. Moreover, it collects the resource consumption information such as CPU usage, memory usage, and processing time of each source code actor. Subsequently, the monitored data are fed into the approximation context engine.

For example, if the application requires an accuracy level of 80% within 10 s, then the approximation engine identifies the Task Processing Path which leads to an 80% level of accuracy within 10 s. The Task Processing Path may include an actor which has parameters such as RGB video, 70% video size, and 20 frame rates. Then, this will trigger an invocation to the actor construction engine, which will create the corresponding source code actor (with the respective parameters). These source code actors will be then deployed into the underlying IoT device and remove the previous source code actors. The monitoring engine will continuously monitor the performance metrics of resources and source code actors and feed this information into the approximation context engine. Therefore, the engine will have real-time resource usage information and is aware of the performance metrics of the source code actors as well (i.e., in terms of execution time).

When the contextual information is changed, the creation of a new set of actors will be triggered that are more suitable for achieving the time-sensitive requirements of the IoT application in the current context. Likewise, whenever the context is changed, the approximation context engine will forward this information to the Approximation engine, then the Approximation engine dynamically identifies the suitable Task Processing Paths. The Task Compiler and Deployment engine stops the execution of previous actors and creates the corresponding actors and deploys the newly created actors to the corresponding IoT device so that the time-sensitive requirement of the IoT application is met.

## 6. Experimental Evaluation

In this section, we describe our experimental testbed setup and evaluation methodology.

The experiments described in this paper were conducted on identical IoT devices with 1GB of RAM with a Linux Ubuntu v18.04 LTS operating system.

To conduct the experiments, we implemented a proof of concept of the framework’s engines using Microsoft Orleans Actor framework [50]. Orleans actor framework is based on the mathematical concurrent computation model called the ‘actor model’. In the actor model, an ‘actor’ is considered as the primitive unit of computation and it communicates with other actors through message passing. Orleans actors are developed to scale in an elastic way and then can be executed on any operating system which has a .NET core installed. Therefore, we decided to implement our framework’s actors as Orleans actors and this facilitated the development of a highly scalable and efficient Task Compiler and Deployment engine on top of the Orleans software framework.

The computing resource including RAM and CPU usage, as well as the breakdown of processing time for each atomic process, have been monitored in real time. The monitoring samples were collected at one-second intervals.

The COVID-19 application algorithm used in the paper has two main components including video pre-processing and video processing (i.e., classification) algorithms.

Video pre-processing is a set of tasks to prepare the video to perform processing algorithms. These tasks can include, but are not limited to, colour profile modification, noise reductions, and so forth. A few examples of video pre-processing tasks are illustrated in Figure 4.

The processing algorithms are responsible for monitoring the enforcement compliance and symptoms monitoring. We utilised ‘Haar Cascade’ object detection algorithm [46,47]. A description of the computer vision algorithms for compliance enforcement and symptom monitoring is outside of the scope of this paper.

### 6.1. Results and Analysis

In this section, the experimental results collected from the experiments will be presented and discussed. The experiments discussed in this paper were performed in a controlled environment using identical IoT devices. We will describe how Sazgar IoT can effectively support time-sensitive COVID-19 citizen compliance monitoring applications.

**Figure 10:**
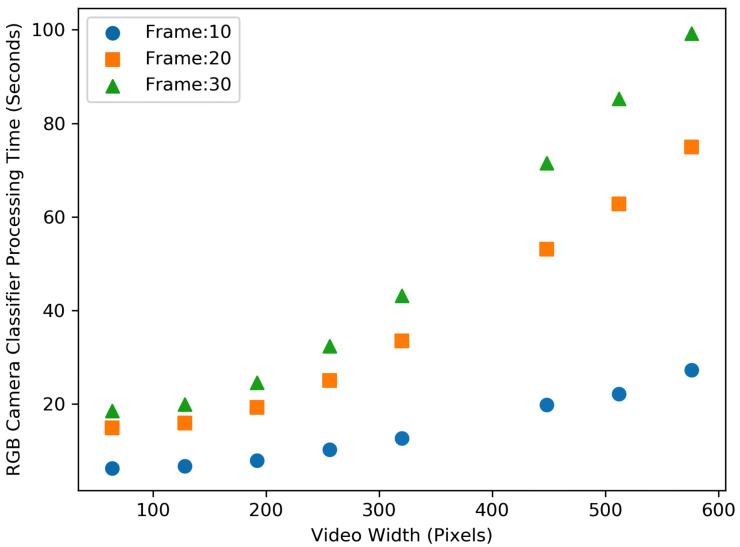
Comparison of the video processing (i.e., classification) time for different RGB video size (width in pixel) and frame rates (per second).

**Figure 11:**
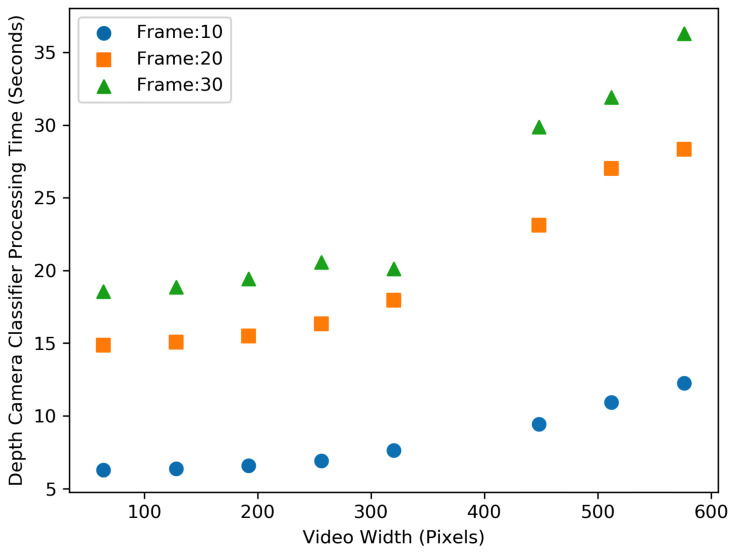
Comparison of the video processing (i.e., classification) time for different depth video size (width in pixel) and frame rates (per second).

Figure 10 and Figure 11 show the processing time for performing the processing (i.e., classification) algorithm (i.e., people detection and counting) on the RGB and depth video data, respectively. As can be seen from the figures, counting the number of people entering and exiting the buses utilising higher resolution video and higher frame rates still need significantly more time to perform the classification. Moreover, RGB video data require two times more time to process the classification on high-resolution videos as compared to depth video. The difference is not very significant for lower-resolution videos.

**Figure 12:**
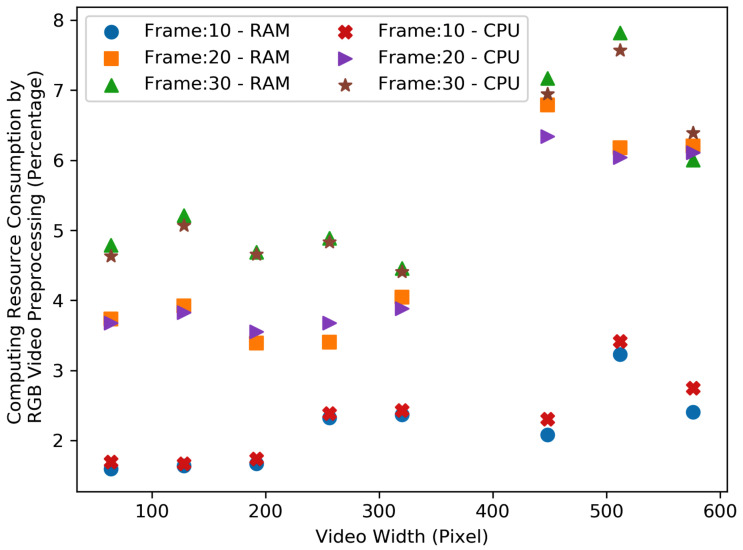
Comparison of the RGB video pre-processing computing resource consumption—RAM and CPU.

**Figure 13:**
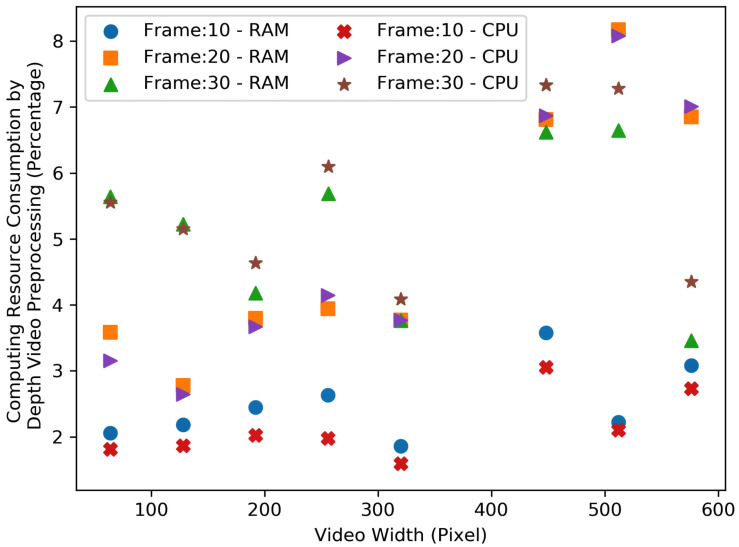
Comparison of the depth video pre-processing computing resource consumption—RAM and CPU.

**Figure 14:**
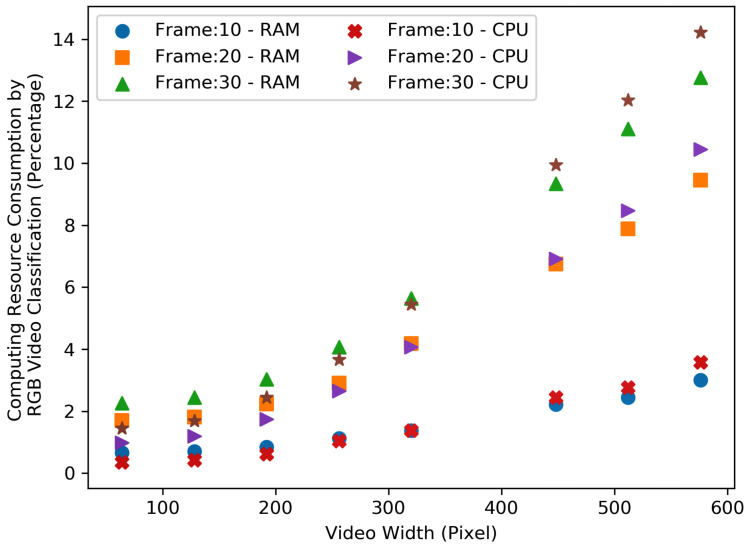
Comparison of the RGB video processing (i.e., classification) computing resource consumption—RAM and CPU.

**Figure 15:**
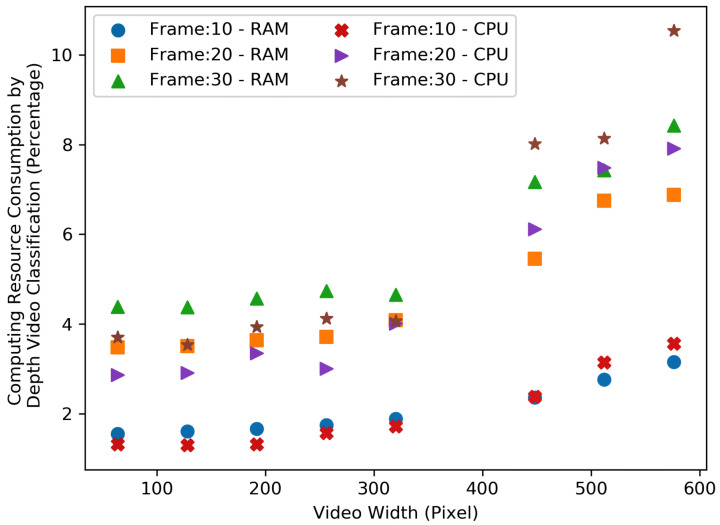
Comparison of the depth video processing (i.e., classification) computing resource consumption—RAM and CPU.

Figure 12 and Figure 13, show the resource consumption for the video pre-processing algorithm. Figure 14 and Figure 15, show the CPU and RAM usage for the processing algorithm performed on RGB and depth video data. Table 3 compares the depth and RGB video pre-processing computing resource consumption. It can be observed that the resource consumption for the processing algorithm is higher than the video pre-processing sub-tasks. In addition, resource consumption follows a linear trend for higher video resolutions in such a way that higher-resolution videos always consume more resources to perform processing algorithms.

**Figure 16:**
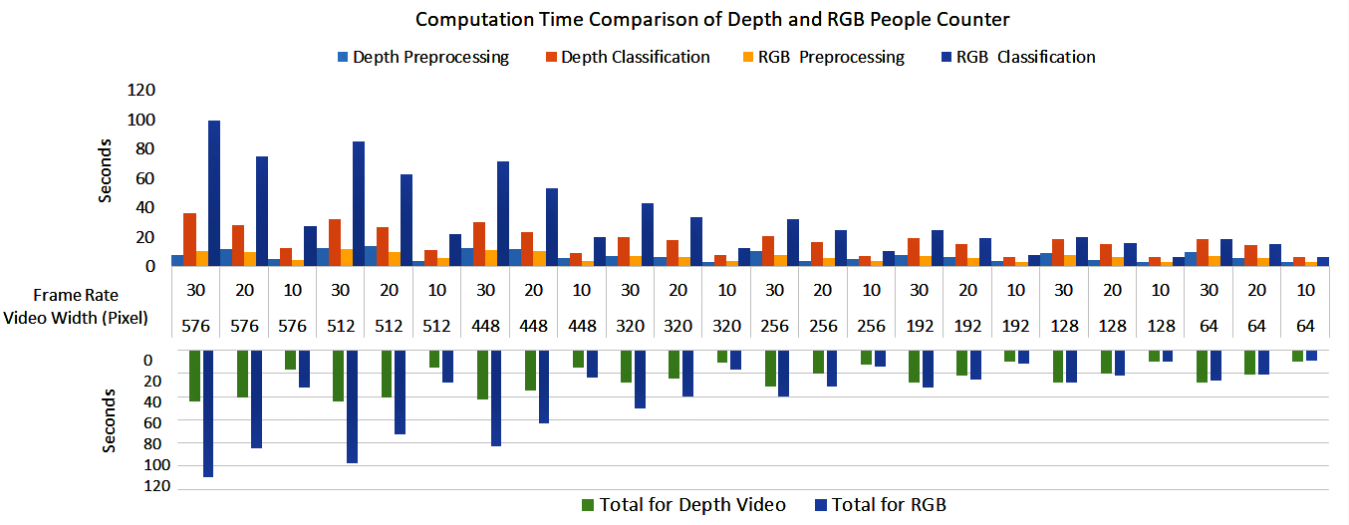
Computation time comparison of depth and RGB people counter.

It is obvious from the figures discussed earlier that the resource consumption and processing time are dependent on the type of videos, video resolution, frame rate, and type of algorithms performed on the data as illustrated in Figure 16.

In this paper, we propose a framework that can understand and take into account these dependencies and use them to perform more effective and efficient IoT data analysis on IoT devices within the time-sensitive requirements while respecting the available computing resources and preferences/requirements from the applications.

**Figure 17:**
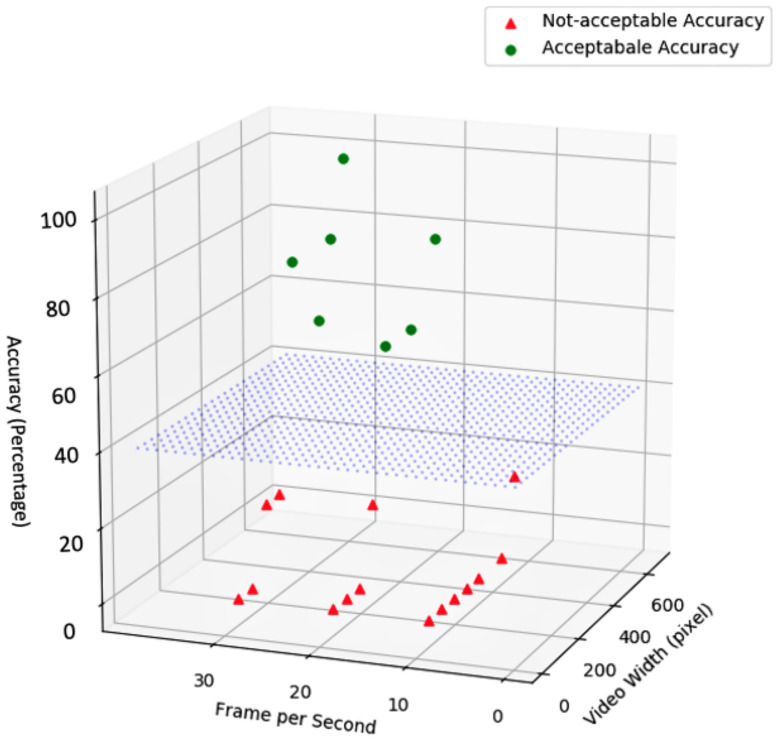
Comparison of the accuracy for different video and frame size.

Figure 17, presents the processing algorithm’s accuracy with respect to frame rate and video resolution (i.e., video width in pixels). The accuracy is represented with a percentage where a higher value means higher accuracy.

The blue surface illustrated in Figure 17 represents an assumed required/preferred accuracy by application. This surface is dynamic and any values above the surface will have sufficient accuracy to be selected by the proposed framework (e.g., in this example, acceptable accuracy is more than 40%; however, in real-world applications, the acceptable accuracy should be more than 70%).

**Figure 18:**
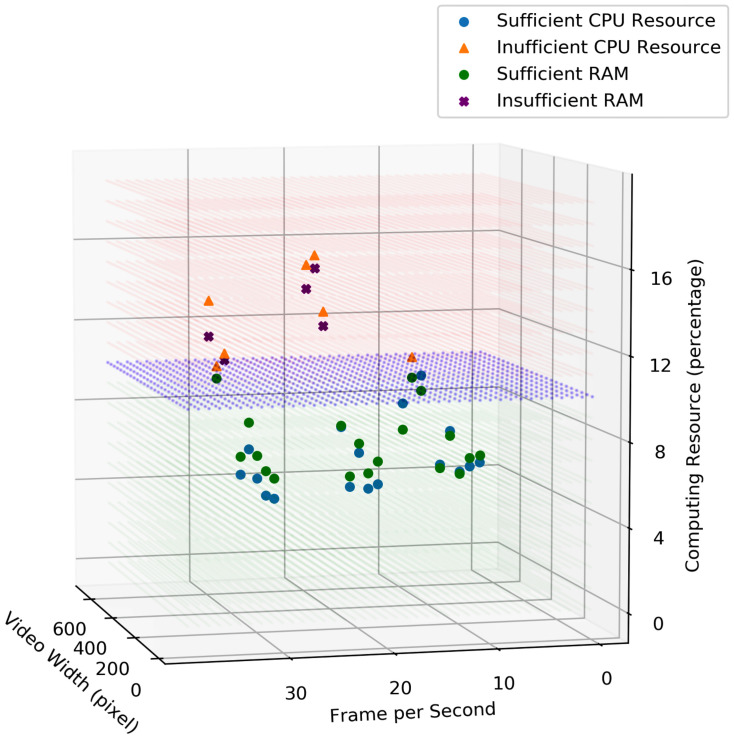
Comparison of the resource consumption for different video and frame size.

Figure 18, represents the RAM and CPU usages for running processing algorithms on the depth video data. Similarly, the blue surface indicates the available/preferred computing resource to perform the processing task (e.g., utilising not more than 10% of the computing resource). The proposed framework chooses the frame rate and video size where the RAM and CPU consumption are both under the blue surface to fulfil the requirements/preferences.

**Figure 19:**
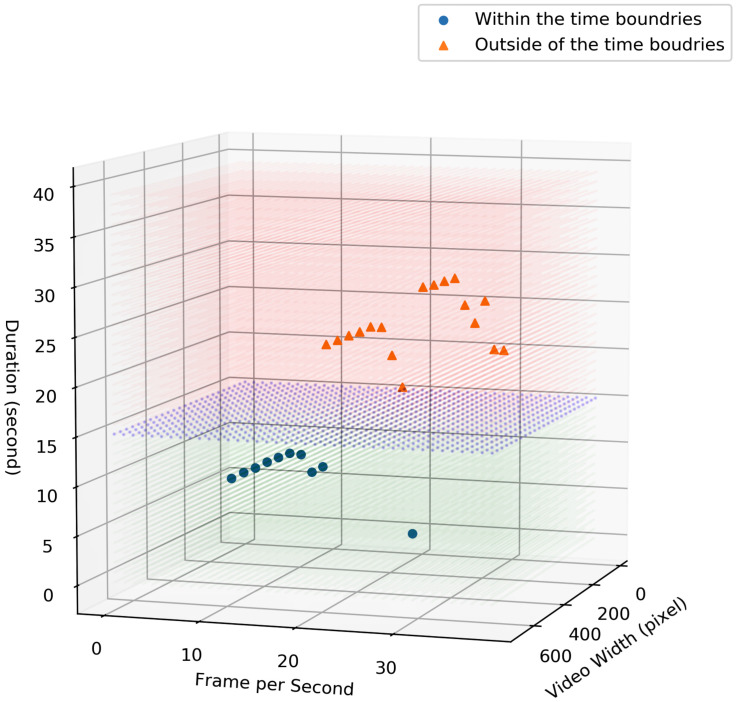
Comparison of the duration of the processes for different video and frame size.

Finally, Figure 19 shows the duration of the process with respect to the video size and frame rates. The blue surface in this figure represents the time-sensitive requirements (e.g., 15 s). As it is illustrated in the figure, any combination of the frame rate and video size in which they can perform the process within the time boundaries can be selected by our proposed framework.

### 6.2. Limitations

While the experimental evaluation demonstrates that the proposed framework fulfils the time-bound and accuracy requirements of the monitoring application, there are certain limitations in the existing approach that need to be addressed. One such limitation is that the experiment was conducted using a fixed number of IoT devices, and it does not account for the potential impact of fluctuations in the number of IoT devices on the accuracy of the approximation results. In addition, the impact of IoT device interoperability on TS-IoT applications must be addressed if other types of sensors are added to IoT devices. Another limitation pertains to data availability. Although data availability is not a limitation of the proposed framework, it is an issue that can impact the accuracy of such time-bound frameworks. Therefore, it is crucial to prioritise data availability in TS-IoT applications that utilise Sazgar IoT to ensure the accuracy of the monitoring process. Furthermore, although device-centric IoT frameworks such as Sazgar IoT can significantly improve the efficiency of TS-IoT applications, it must also be considered that they compromise cloud-specific IoT capabilities such as scalability, advanced analytics, data storage and management, integration with external services, and remote monitoring and management. Another notable drawback of the suggested framework is that the approximation technique may not be suitable for IoT applications that necessitate a 100 percent accuracy rate. This implies that there is a trade-off between the time sensitivity and accuracy sensitivity of the IoT application, which must be taken into account when choosing between device-to-cloud or device-centric architectures. While device-centric frameworks such as Sazgar can significantly enhance processing speed, they may also result in a lower accuracy rate compared to cloud-based architectures.

## 7. Conclusions and Future Work

As the IoT brought about unprecedented changes in modern society, the need for collecting and processing data efficiently has become more important than ever. There exist many approaches to data harvesting and processing but many rely on highly resourceful devices such as those in the cloud. While many applications benefit from outsourcing data processing, time-sensitive applications that are sensitive to delays can benefit from processing the collected data locally (trading off computational resources with time). In this paper, we proposed an IoT device-centric data analysis framework for time-sensitive monitoring of COVID-19 lockdown compliance and symptoms. This framework can take into account contextual information and perform approximation to enable the processing of the data on the IoT device itself while satisfying the application-specified outcomes and time-sensitive requirements. The contextual information (Computing Resource Context and Application Requirement Context) and approximation technique subsequently enabled IoT end devices to intelligently perform several tasks relevant to monitoring COVID-19 lockdown compliance and symptoms on IoT devices without needing access to cloud or edge computing resources. It must be noted that when using the proposed framework in real-world scenarios, the ideal activity detection accuracy should be more than 70%.

Future work involves investigating accuracy-based decision making in such a way that the framework will dynamically switch to a processing path that delivers the best accuracy in any given context. Another avenue to explore would be the use of a granular and context-aware disclosure technique as proposed in [51] to maintain privacy-preserving in our COVID-19 application. This technique can dynamically maintain data security utilising the contextual information described in this paper. Other research directions include deploying rate adaptation intelligence at video-enabled end devices [52], implementing latency-reducing techniques [53], incorporating network-based feedback (bandwidth/delay measurements and predictions) as contextual information, and experimenting with advanced data analytic techniques and emerging IoT-based communication technologies.

## Figures and Tables

**Table 1:** Comparison of the state-of-the-art in IoT solutions meeting the IoT application requirements.

	QoS	Energy Consumption	Scalability	Latency	Security	Interoperability	Reliability	Cost
[8]	✓	✓		✓				✓
[16]	✓			✓				✓
[17]			✓	✓			✓	
[18]		✓		✓	✓			✓
[9]	✓		✓	✓	✓			✓
[19]							✓	✓
[10]	✓			✓		✓	✓	✓
[20]	✓			✓				✓
[21]			✓				✓	
[22]	✓	✓		✓			✓	✓

**Table 2:** Detection time-bounds and corresponding accuracy for citizen activities and symptoms.

Citizen Activities and Symptoms in Public Places	Detection Time-Bound	Detection Accuracy
Social distancing	Less than 5 s	More than 65%
Wearing masks	Less than 30 s	More than 80%
Sanitising hands	Less than 5 s	More than 50%
Curfew	Less than 1 h	More than 95%
Visiting hotspots	Less than 30 min	More than 85%
People counting	Less than 3 s	More than 40%
Symptoms monitoring	Less than 30 s	More than 90%
Contact tracing	Less than 1 h	More than 95%

**Table 3:** Comparison of the depth and RGB video pre-processing computing resource consumption.

	Depth						RGB					
	Frame 10		Frame 20		Frame 30		Frame 10		Frame 20		Frame 30	
Video Width	RAM %	CPU %	RAM %	CPU %	RAM %	CPU %	RAM %	CPU %	RAM %	CPU %	RAM %	CPU %
64	2.06	1.81	3.59	3.17	5.62	5.53	1.58	1.72	3.74	3.69	4.76	4.65
128	2.19	1.86	2.78	2.65	5.20	5.15	1.63	1.70	3.94	3.84	5.19	5.07
192	2.46	2.03	3.79	3.67	4.16	4.64	1.67	1.75	3.41	3.58	4.68	4.70
256	2.65	1.98	3.96	4.17	5.67	6.12	2.33	2.41	3.43	3.69	4.89	4.84
320	1.86	1.61	3.77	3.77	3.76	4.07	2.37	2.44	4.05	3.89	4.45	4.42
448	3.59	3.07	6.84	6.88	6.59	7.35	2.08	2.33	6.82	6.34	7.15	6.93
512	2.21	2.11	8.20	8.08	6.62	7.28	3.23	3.43	6.19	6.05	7.81	7.58
576	3.08	2.73	6.86	7.03	3.44	4.36	2.41	2.75	6.23	6.13	5.98	6.41
